# Hijacking of the O-GlcNAcZYME complex by the HTLV-1 Tax oncoprotein facilitates viral transcription

**DOI:** 10.1371/journal.ppat.1006518

**Published:** 2017-07-24

**Authors:** Damien Groussaud, Mostafa Khair, Armelle I. Tollenaere, Laetitia Waast, Mei-Shiue Kuo, Marianne Mangeney, Christophe Martella, Yann Fardini, Solène Coste, Mouloud Souidi, Laurence Benit, Claudine Pique, Tarik Issad

**Affiliations:** 1 INSERM, U1016, Institut Cochin, Paris, France; 2 CNRS, UMR8104, Paris, France; 3 Université Paris Descartes, Sorbonne Paris Cité, Paris, France; University of Illinois at Chicago College of Medicine, UNITED STATES

## Abstract

The viral Tax oncoprotein plays a key role in both Human T-cell lymphotropic virus type 1 (HTLV-1)-replication and HTLV-1-associated pathologies, notably adult T-cell leukemia. Tax governs the transcription from the viral 5’LTR, enhancing thereby its own expression, via the recruitment of dimers of phosphorylated CREB to cAMP-response elements located within the U3 region (vCRE). In addition to phosphorylation, CREB is also the target of O-GlcNAcylation, another reversible post-translational modification involved in a wide range of diseases, including cancers. O-GlcNAcylation consists in the addition of O-linked-*N*-acetylglucosamine (O-GlcNAc) on Serine or Threonine residues, a process controlled by two enzymes: *O*-GlcNAc transferase (OGT), which transfers O-GlcNAc on proteins, and *O*-GlcNAcase (OGA), which removes it. In this study, we investigated the status of O-GlcNAcylation enzymes in HTLV-1-transformed T cells. We found that OGA mRNA and protein expression levels are increased in HTLV-1-transformed T cells as compared to control T cell lines while OGT expression is unchanged. However, higher OGA production coincides with a reduction in OGA specific activity, showing that HTLV-1-transformed T cells produce high level of a less active form of OGA. Introducing Tax into HEK-293T cells or Tax-negative HTLV-1-transformed TL-om1 T cells is sufficient to inhibit OGA activity and increase total O-GlcNAcylation, without any change in OGT activity. Furthermore, Tax interacts with the OGT/OGA complex and inhibits the activity of OGT-bound OGA. Pharmacological inhibition of OGA increases CREB O-GlcNAcylation as well as HTLV-1-LTR transactivation by Tax and CREB recruitment to the LTR. Moreover, overexpression of wild-type CREB but not a CREB protein mutated on a previously described O-GlcNAcylation site enhances Tax-mediated LTR transactivation. Finally, both OGT and OGA are recruited to the LTR. These findings reveal the interplay between Tax and the O-GlcNAcylation pathway and identify new key molecular actors involved in the assembly of the Tax-dependent transactivation complex.

## Author summary

Human T-cell lymphotropic virus type 1 (HTLV-1) is the only human retrovirus associated to a cancer. Indeed, HTLV-1 is responsible for adult T-cell leukemia, an aggressive malignant proliferation of CD4+ T lymphocytes. The regulatory protein Tax governs HTLV-1 transcription from the 5’LTR, driving expression of all viral proteins, including itself, at the exception of the antisense product HBZ. Besides this critical role in HTLV-1 expression, Tax acts as an oncoprotein able to induce T-cell immortalization *in vitro* and tumor formation in mice. In this study, we report that Tax interacts with the O-GlcNAczyme OGT/OGA complex that catalyzes O-GlcNAcylation, a post-translational modification often deregulated in cancers. We found that Tax interacts with the OGT/OGA complex and inhibits the activity of OGA, increasing thereby cellular O-GlcNAcylation. Strikingly, we found that O-GlcNAcylation of CREB, the cellular transcription factor recruited by Tax on the viral promoter, is increased in a Tax-dependent manner. Moreover, increased CREB O-GlcNAcylation strongly enhances Tax-induced LTR transactivation as well as CREB binding to the viral promoter. Finally, both OGT and OGA are part of the transactivation complex. These findings shed new light on the mechanism of Tax-dependent LTR transactivation and may open the way to new molecular interventions targeting HTLV-1 expression.

## Introduction

Human T-lymphotropic virus type 1 (HTLV-1) is the only retrovirus associated to a cancer in humans. HTLV-1 is indeed the etiologic agent of adult T-cell leukemia/lymphoma (ATLL), a very aggressive malignant proliferation of CD4+ T lymphocytes, which appears in 2–5% of infected individuals (reviewed in [1]). In addition, HTLV-1 is also associated with various inflammatory disorders, including HTLV-1-associated myelopathy/tropical spastic paraparesis (HAM/TSP) [2].

The oncogenic power of HTLV-1 is due in large part to the properties of the viral oncoprotein Tax. Tax is a powerful inducer of T-cell proliferation through its ability to activate a broad range of cellular promoters, promote cell cycle and inhibit apoptosis and repair machineries (reviewed in [3]). As a consequence, Tax has been shown to induce immortalization of primary T cells *in vitro* [4] as well as tumor formation in transgenic animals [5]. Tax is also critical for HTLV-1 gene expression by virtue of its capacity to transactivate the 5’ LTR that controls the transcription of all HTLV-1 structural, enzymatic and regulatory genes, including Tax itself, and auxiliary genes with the exception of the antisense product HBZ [6].

The transactivation of the 5’LTR depends on Tax interaction with the cellular transcription factor cAMP response element binding protein (CREB) that, together with Tax, binds to three conserved copies of a cyclic AMP-response element (CRE) located in the LTR U3 region (viral CRE/vCRE). CREB-mediated activation of cellular promoters has been shown to critically depend on CREB phosphorylation at Ser133 [7, 8]. It was initially proposed that CREB phosphorylation was dispensable in the context of Tax transactivation of the HTLV-1 promoter [9, 10]. However, further studies demonstrated on the one hand that the transactivation complex contains Ser133-phosphorylated CREB and on the other hand, that Tax is able to increase CREB phosphorylation [11–13]. The binding of Tax/CREB complexes to the vCRE then allows the recruitment of the CREB-Regulated Transcription Coactivator/Transducer Of Regulated CREB-Binding Protein (CRTC/TORC) [14], the CREB binding protein (CBP) [15] and CBP-associated factor (p/CAF) [16] general co-activators and ultimately, of components of the basal transcription machinery (reviewed in [17]).

O-GlcNAcylation is a reversible post-translational modification [18] that has been shown to regulate stability, sub-cellular localisation and/or activity of a large set of proteins, notably transcription factors or co-factors [19], including CREB [20–22]. O-GlcNAcylation consists in the addition of N-acetyl glucosamine (GlcNAc) on Serine and Threonine residues. Only a unique couple of enzymes controls O-GlcNAc level on proteins: OGT (O-GlcNAc transferase), which adds the GlcNAc motif on proteins, and OGA (O-GlcNAcase), which removes it [19]. OGT and OGA are known to be physically associated in a molecular complex (the O-GlcNAczyme complex), and this association was shown previously to be important for their regulatory activity on cell signaling and transcriptional processes [23]. Numerous studies have reported alterations in OGT, OGA and O-GlcNAc levels in solid tumors as well as hematopoietic cancers [24]. O-GlcNAcylation may promote tumor development through perturbation of signalling pathways and cell cycle regulators [24, 25]. In addition, major oncogenic factors were shown to be directly O-GlcNAcylated [24, 25]. Finally, O-GlcNAcylation has been recently recognized as a novel epigenetic mark (reviewed in [26]).

O-GlcNAcylation of CREB was initially described in rat brain [20]. Serine 40 of CREB was identified as a major O-GlcNAcylation site and found to function as a negative signal by preventing CREB association with CRTC/TORC [21]. CREB can be simultaneously O-GlcNAcylated at Ser40 and phosphorylated at Ser133 and indeed, CREB O-GlcNAcylation was shown to preferentially occur on the population of Ser133-phosphorylated CREB [21, 22].

In this study, we explore for the first time the status of O-GlcNAcylation in HTLV-1-transformed T cells. By using a combination of BRET, enzymatic and biochemical assays, we report that the HTLV-1 Tax protein binds to the O-GlcNAczyme complex, blocks the activity of OGA and increases total O-GlcNAcylation in both adherent cells and HTLV-1-transformed T cells. Moreover, we show on the one hand that Tax increases CREB O-GlcNAcylation and on the other hand that increasing O-GlcNAcylation through OGA inhibition enhances both Tax-induced LTR transactivation and CREB recruitment to the promoter. We also report that in contrast to wild-type CREB, the CREB S40A mutant fails to enhance Tax-mediated LTR transactivation. Finally, we show that both OGT and OGA are recruited to the HTLV-1 LTR. These findings identify new functional interacting partners of Tax and shed new light on the composition of the transactivation complex assembled by Tax on the HTLV-1 5’ LTR promoter.

## Results

### HTLV-1-transformed T cells accumulate a less active form of OGA

**Fig 1:**
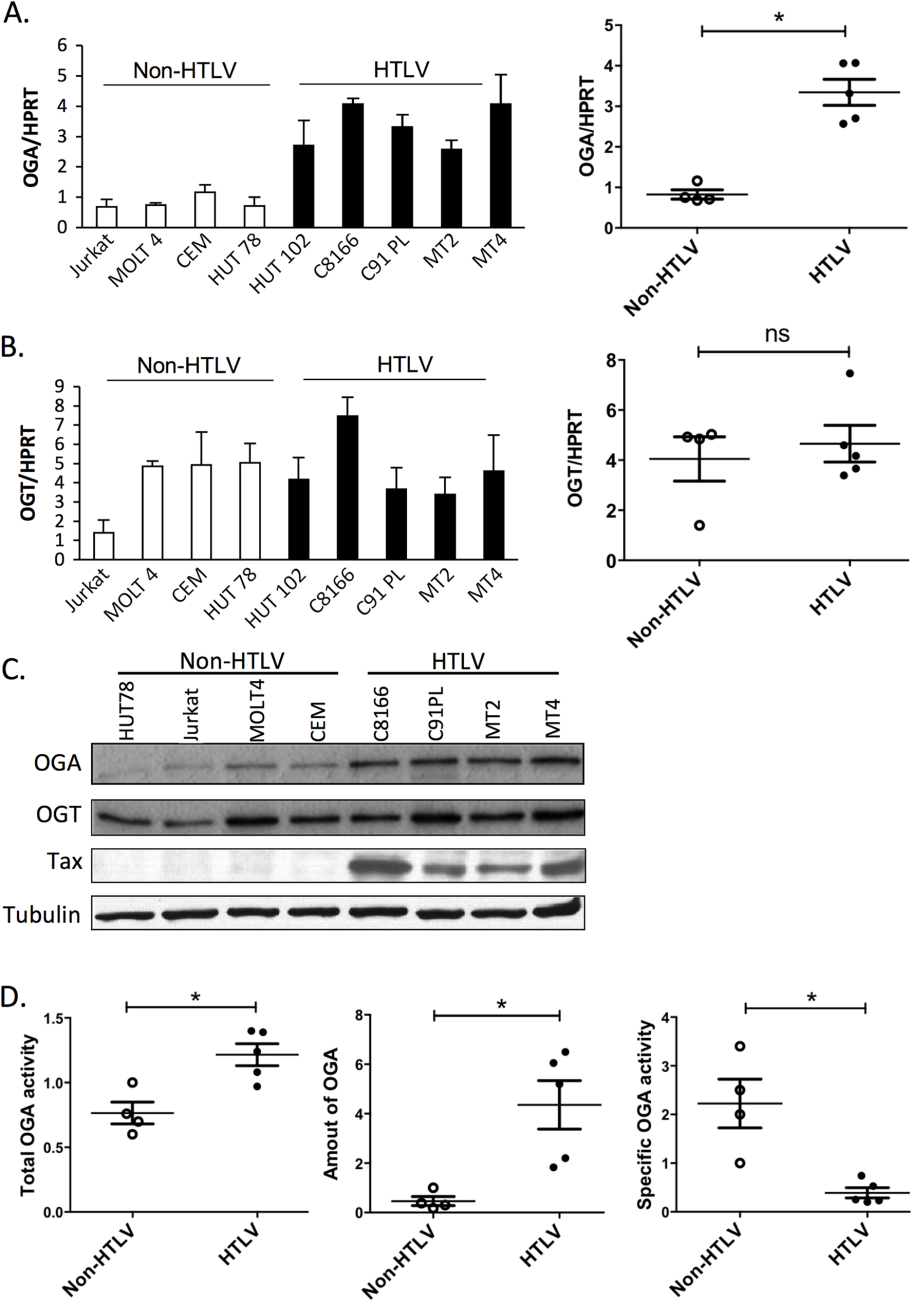
OGA and OGT status in HTLV-1-transformed T cells and control transformed T cells. (**A, B**) RT-qPCR experiments comparing the amount of OGA (**A**) or OGT (**B**) mRNA in non-HTLV-1 (white bars) or in HTLV-1-transformed T cells (black bars) normalized to the level of HPRT mRNA. Results are means ± SEM of two independent experiments performed in triplicates. Statistical analyses are shown in right panels. (**C**) Western blot experiments showing the amount of OGA, OGT, Tax and tubulin in non-HTLV-1 or in HTLV-1-transformed T cells extracts. (**D**) OGA activity in non-HTLV-1 (empty circle) or HTLV-1 (black circle) transformed T cell extracts. Total OGA activity (left panel) was normalized to the OGA protein level in the extract determined by densitometric analysis of the OGA band detected on western-blot (middle panel), in order to estimate OGA specific activity (right panel). Results correspond to 3 independent experiments. Statistical significance was analyzed using the student’s t test (ns: not significant; *: p<0.05).

To determine the status of O-GlcNAcylation in T cells upon HTLV-1-induced transformation, the levels of OGT and OGA were compared between T cells transformed or not by HTLV-1. Greater level of OGA mRNA was found in four HTLV-1-transformed T cell lines, compared to four non-HTLV-1 transformed T cells (Fig 1A), whereas OGT mRNA expression was not affected (Fig 1B). To determine whether increased OGA mRNA expression could be related to the activated phenotype of HTLV-1 transformed T cells, we evaluated the levels of OGA and OGT mRNA upon T-cell activation. In contrast to HTLV-1-induced transformation, activation of peripheral blood mononuclear cells with PHA and IL-2 strongly reduced the level of OGA mRNA, while increasing OGT mRNA expression (S1 Fig). Hence, HTLV-1-induced T-cell transformation and T-cell activation differentially modulate OGA and OGT mRNA expression. As shown in Fig 1C, western blot analysis confirmed increased OGA protein expression with no change in OGT protein expression in HTLV-1-transformed compared to non-HTLV-1 transformed T cells.

The enzymatic activity of OGA in each T cell line was then quantified. Cells were lysed and equal amounts of total proteins were used to measure either total OGA enzymatic activity or OGA protein level (Fig 1D). A statistically significant increase in total OGA activity (p = 0.0317) was found in HTLV-1-transformed T cells as compared to control transformed T cells (Fig 1D, left panel). Because OGA protein expression level was higher in HTLV-1-transformed T cells, OGA activities were normalized to the amount of OGA protein present in each assay, determined by quantification of the signal obtained by western-blotting using the same cell extracts (Fig 1D, middle panel). To validate this procedure, we verified that a linear relationship exists between OGA activity and the OGA signal obtained by western-blot (S2 Fig). When corrected for OGA expression levels, OGA specific activity was much lower (p = 0.0159) in HTLV-1-transformed T-cells than in control transformed T cells (Fig 1D, right panel).

These findings show that OGT and OGA expression levels are differentially affected by HTLV-1 transformation. They also show that OGA production is increased at both mRNA and protein levels in HTLV-1-transformed T cells but that the activity of the enzyme is impaired in these cells.

### Tax inhibits OGA activity and increases cellular O-GlcNAcylation

**Fig 2:**
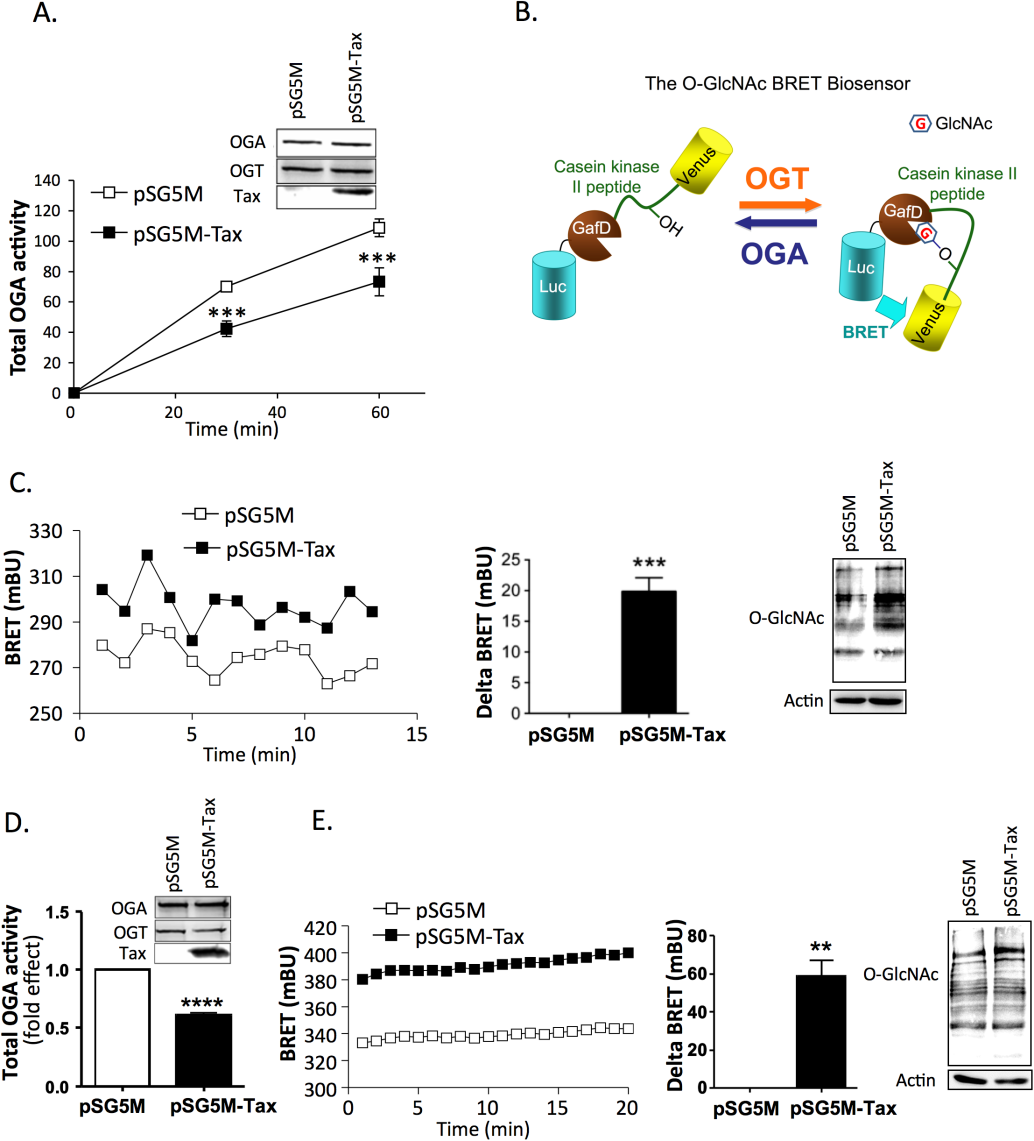
Effect of Tax on OGA activity and O-GlcNAcylation. (**A**) Total OGA activity was measured at two different time-points in extracts from TL-om1 T cells transfected with either the control or Tax plasmid. Results are means ± SEM of 4 independent experiments. The insert shows the expression of OGA, OGT and Tax in the cell extracts. (**B**) The BRET O-GlcNAc-biosensor is composed of Rluc8 fused to the fimbrial adhesin lectin domain GafD, a known OGT substrate peptide derived from casein kinase II placed between two flexible linkers (GGSGG), followed by the Venus variant of the yellow fluorescent protein (Adapted from [27]). (**C**) TL-om1 cells were transfected with the O-GlcNAc BRET biosensor and either the control or Tax plasmid. BRET experiments were performed 24h after transfection. BRET measurements were started 5 min after the addition of coelenterazine. Left panel: typical BRET experiment. Middle panel: mean delta BRET (increased BRET signal induced by Tax expression). Results are means ± SEM of 3 independent experiments. Right panel: level of O-GlcNAc proteins evaluated by western-blotting with an anti-O-GlcNAc antibody. (**D**) Total OGA activity was measured in extracts from HEK-293T cells transfected with either the control or Tax plasmid. Cells were extracted and OGA activity was measured at 30 min. Results are means ± SEM of 3 independent experiments. The insert shows the expression of OGA, OGT and Tax in the cell extracts. (**E**) HEK-293T cells were transfected with the O-GlcNAc BRET biosensor and either the control or Tax plasmid. BRET experiments were performed 48h after transfection. BRET measurements were started 5 min after the addition of coelenterazine. Left panel: typical BRET experiment. Middle panel: mean delta BRET (increased BRET signal induced by Tax expression). Results are means ± SEM of 3 independent experiments. Right panel: level of O-GlcNAc proteins evaluated by western-blotting with an anti-O-GlcNAc antibody. Statistical significance was calculated using the student’s t test (**: p<0.01; ***: p<0.001; ****: p<0.0001).

The HTLV-1 Tax protein is capable of interacting with and deregulating numerous cellular proteins and machineries [3]. We therefore evaluated the impact of Tax on OGA activity using a Tax-negative HTLV-1 transformed T cell line (TL-om1), which allowed us to study Tax activity in an HTLV-1-transformed T cell context. A Tax expressor plasmid was transfected into TL-om1 T cells and OGA activity was measured 24 hours post-transfection. We observed that Tax-expressing TL-om1 T cells exhibited lower OGA activity than TL-om1 T cells transfected with the control plasmid (Fig 2A). This reduction in OGA activity was not due to a change in OGA expression level (Fig 2A, insert).

In order to determine whether Tax-induced OGA inhibition was associated with a change in O-GlcNAcylation, we developed a BRET biosensor based on a previously described FRET O-GlcNAc biosensor (Fig 2B)[27]. This BRET O-GlcNAc biosensor is composed of Rluc8 fused to a lectin domain (GafD), a known OGT substrate peptide derived from casein kinase II, followed by the Venus variant of the yellow fluorescent protein. Upon O-GlcNAcylation, the casein kinase peptide binds to the lectin, resulting into a conformational change detected as an increased BRET signal (Fig 2B). We observed higher BRET signal in TL-om1 cells expressing Tax compared to control cells (Fig 2C, left panel and statistical analysis of Tax-induced delta BRET in middle panel). This result was confirmed by western-blotting with an anti-O-GlcNAc antibody, which showed increased O-GlcNAcylation of proteins in Tax-transfected cells (Fig 2C, right panel).

Since TL-om1 T cells still express the viral antisense product HBZ, we investigated the effect of Tax in an HTLV-1-independent context. In transfected HEK-293T cells, Tax expression also resulted in a marked reduction in OGA enzymatic activity, as compared to control cells (Fig 2D). Again, this effect was not due to a change in OGA expression level (Fig 2D, insert). In contrast to OGA, OGT activity was not affected by Tax expression (S3 Fig). Inhibition of OGA activity coincided with a significant increase in the BRET signal of the biosensor (Fig 2E left panel and statistical analysis of Tax-induced delta BRET in middle panel). An increase in O-GlcNAcylation level of HEK-293T cell proteins was also detected by western-blotting using the anti-O-GlcNAc antibody (Fig 2E, right panel).

These results suggest that Tax inhibits OGA activity independently of the HTLV-1 context, and that this inhibition results in increased cellular O-GlcNAcylation.

### Tax interaction with the O-GlcNAczyme complex

**Fig 3:**
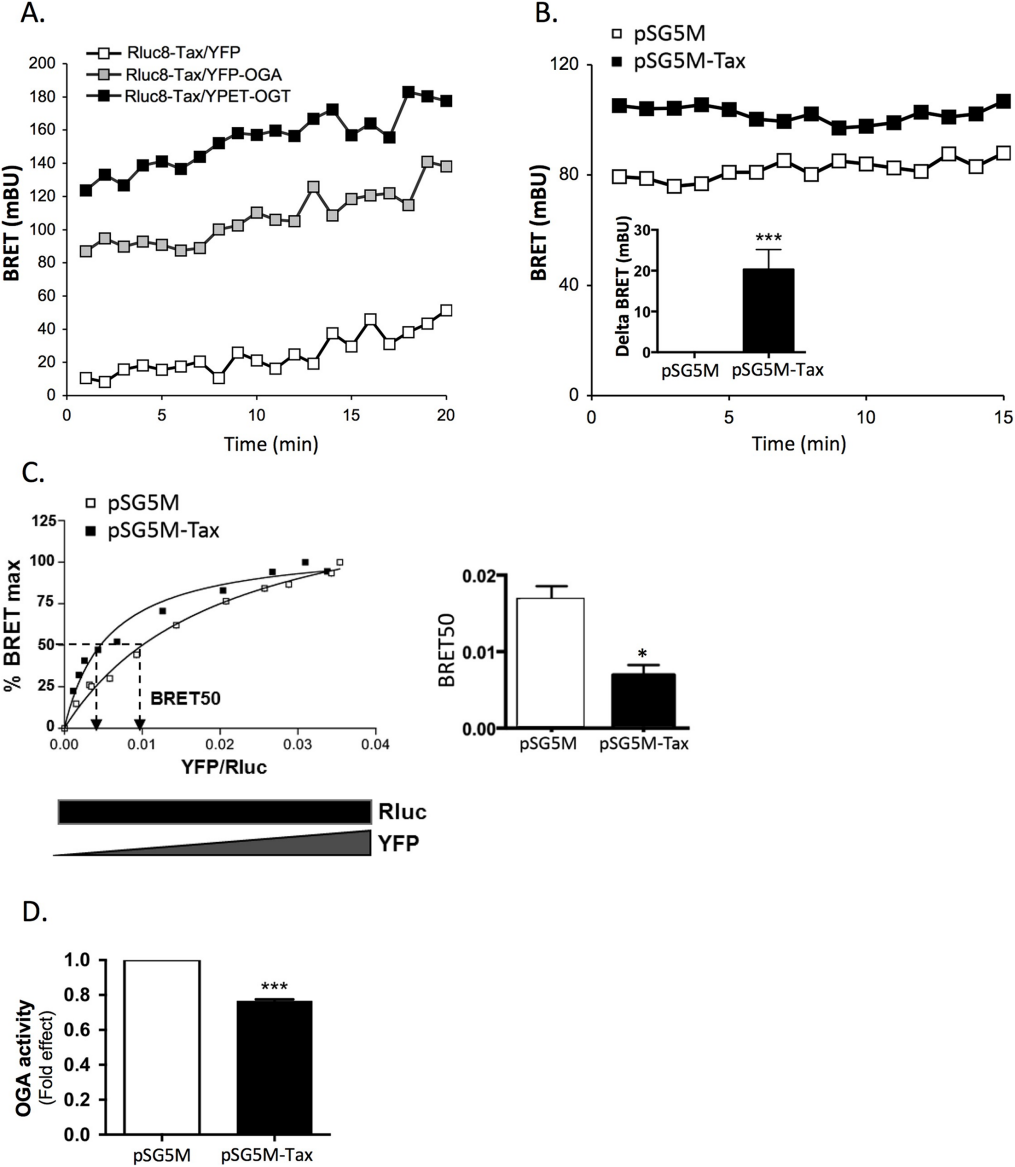
Tax interacts with the O-GlcNAczyme complex. (**A**) HEK-293T cells were co-transfected with Rluc8-Tax and YFP, YFP-OGA or YPET-OGT. BRET experiments were performed 48h after transfection. BRET measurements were started 5 min after the addition of coelenterazine. (**B**) HEK-293T cells were co-transfected with YFP-OGA and Rluc8-OGT and either the control or Tax plasmid, and BRET experiments were performed 48h after transfection. Tax-induced increase in BRET is shown in the insert. (**C**) For BRET saturation assays, HEK-293T cells were co-transfected with a constant amount of cDNA coding for Rluc8-OGT (300 ng/well) and increasing amount of cDNA coding for OGA-YFP (10 to 1000 ng/well) and either the control or Tax plasmid. BRET signal, luciferase and fluorescence levels were measured 48h post-transfection. Left panel: A typical BRET saturation experiment is shown. BRET signals were plotted as a function of the ratio of YFP-OGA fluorescence to Rluc8-OGT luminescence. The curves were fitted using non-linear regression equation assuming a single binding site (GraphPad Prism). Right panel: Effect of Tax expression on the BRET50 derived from BRET saturation curves using GraphPad Prism. (**D**) HEK-293T cells were co-transfected with the control or Tax plasmid, Rluc8-OGT, YFP-OGA, or both. After immunoprecipitation with the anti-OGT antibody, the OGA activity present in the immune complex was measured and normalized to the amount of YFP fluorescence recovered in the immune complex. Results are mean ± SEM of 3 to 4 independent experiments. Statistical significance was analyzed using the Student’s t test (*: p<0.05; **: p<0.01; ***: p<0.001).

OGT and OGA have been previously shown to form a molecular complex, referred to as the O-GlcNAczyme, which plays an important role in their biological functions [23]. To determine whether Tax may alter O-GlcNAcylation by interacting with this complex, we first evaluated by BRET the interaction of Tax with either OGA or OGT. HEK-293T cells were transfected with a cDNA coding for a luciferase-tagged Tax (Rluc8-Tax) together with YPET-OGT, YFP-OGA, or YFP alone. Western blot analysis showed correct expression of each of these fusion proteins at their expected molecular weights (S4 Fig). A much higher BRET signal was observed with YPET-OGT or YFP-OGA than with YFP, indicating a specific interaction of Tax with the O-GlcNAc cycling enzymes (Fig 3A).

We then studied the effect of Tax expression on the formation of the OGT/OGA complex by BRET in HEK-293T cells co-transfected with OGT-Rluc and OGA-YFP constructs. As shown in Fig 3B, such complex could be readily detected as a BRET signal between OGT-Rluc and OGA-YFP. A higher BRET signal was found upon Tax expression, suggesting that Tax modulates OGT/OGA interaction (Fig 3B and statistical analysis of Tax-induced delta BRET in the insert). To further analyze the effect of Tax on OGT/OGA interaction, BRET saturation assays were performed (Fig 3C). This analysis permits to determine whether a change in BRET signal between two partners corresponds to an increased affinity between the two partners (reflected by decreased BRET_50_) [28] or, rather, a conformational change within the complex that modifies the relative orientation between the luciferase and the YFP, resulting in a higher efficiency of energy transfer, without change in BRET_50_ [29]. Analysis of the saturation curves using Prisme software indicated that Tax expression reduces the BRET_50_ (Fig 3C left panel and statistical analysis of BRET_50_ in the right panel), suggesting that Tax may regulate O-GlcNAcylation by increasing the affinity between OGA and OGT.

We next measured the enzymatic OGA activity in the OGT/OGA complex after immunoprecipitation of OGT. HEK-293T cells were co-transfected with OGT-Luc, OGA-YFP and either the Tax or control plasmid. OGA activity was measured on the immune complex and normalized to YFP fluorescence of the precipitated proteins. We found that Tax significantly reduced the activity of OGA co-immunoprecipitated with OGT (Fig 3D).

Taken together, these data support the notion that Tax regulates O-GlcNAcylation by modulating OGT/OGA interaction, resulting in inhibition of OGA activity in the O-GlcNAczyme complex.

### Pharmacological inhibition of OGA enhances LTR activation in Tax expressing cells

**Fig 4:**
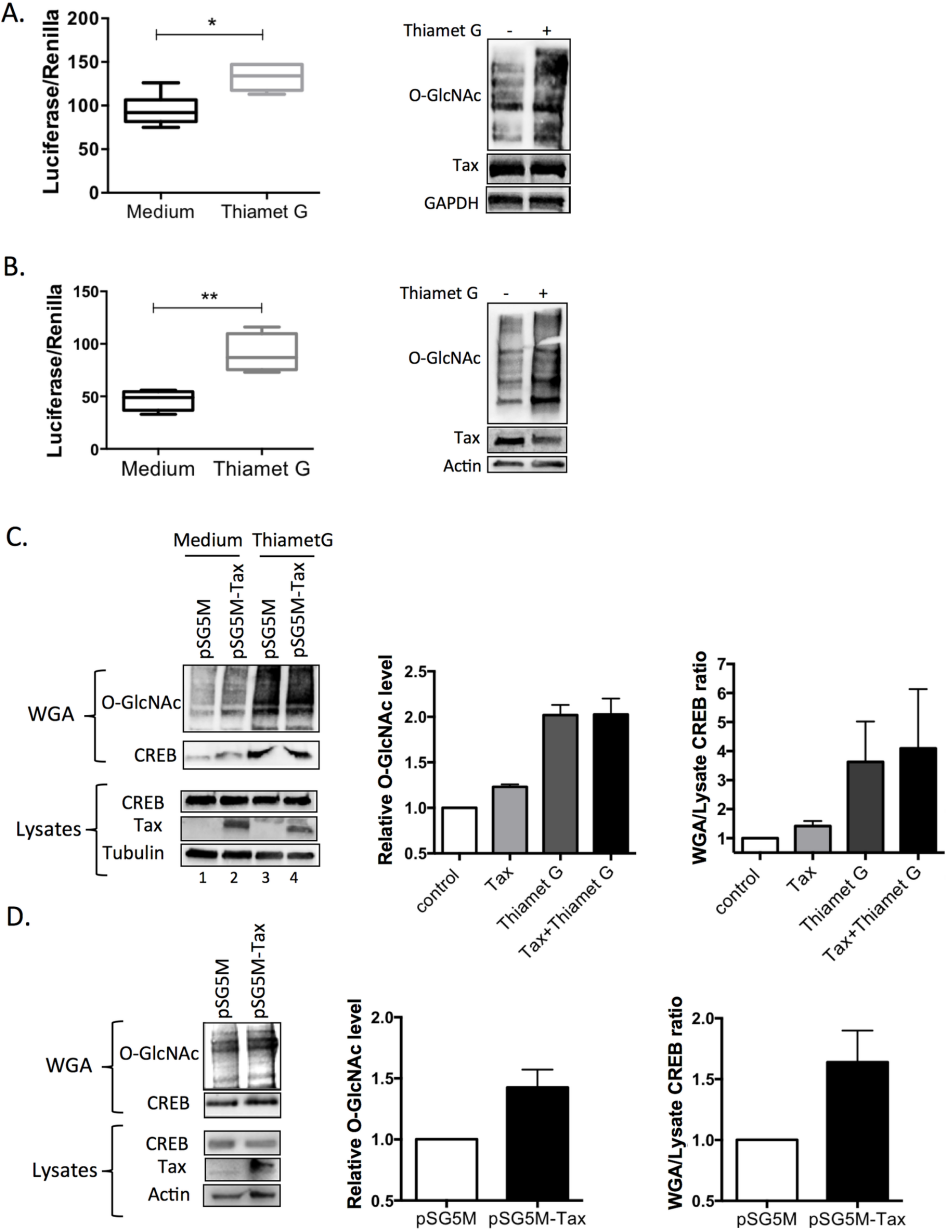
Effect of OGA inhibition on LTR activation and CREB O-GlcNAcylation in Tax expressing cells. (**A, B**) Effect of OGA inhibition on Tax-mediated LTR transactivation in (**A**) C8166 T cells or (**B**) HEK-293T cells. Left panels: Cells were transfected with the U3R-LTR-luciferase and pRL-TK plasmid and in the case of HEK-293T cells, the Tax plasmid, and were incubated or not with the OGA inhibitor Thiamet G for 24h. Luciferase production was then measured using the dual luciferase assay. Results are means ± SEM of 4 independent experiments performed in duplicates. Right panels: level of O-GlcNAcylation and Tax expression in each condition. (**C**) Effect of Tax on CREB O-GlcNAcylation in HEK-293T cells. HEK-293T cells were transfected with either the control or Tax plasmid and treated or not with the OGA inhibitor Thiamet G. Cell extracts were prepared two days post-transfection. Left panel: total proteins (lysates) or WGA-bound proteins (WGA) were separated by SDS-PAGE and blotted with either an anti-O-GlcNAc or an anti-CREB antibody. Tax expression in cell lysates is also shown. Middle and right panel: Total O-GlcNAc signal (middle panel) or WGA/lysate ratio for CREB signal (right panel) in two independents experiments. (**D**) Effect of Tax on CREB O-GlcNAcylation in TL-om1 T cells. TL-om1 T cells were transfected with either the control or Tax plasmid. Left panel: Total proteins (lysates) or WGA-bound proteins (WGA) were separated by SDS-PAGE and blotted with either an anti-O-GlcNAc or anti-CREB antibody. Tax expression in cell lysates is also shown. Middle and right panels: Total O-GlcNAc signal (middle panel) or WGA/lysate ratio for CREB signal (right panel) in two independents experiments.

We next studied the impact of increasing O-GlcNAcylation by using the specific OGA inhibitor Thiamet G on the activity of the HTLV-1 LTR in Tax expressing cells. C8166 T cells transfected with the HTLV-1-U3R-Firefly Luciferase construct (U3R-Luc) and the pRL-TK normalisation plasmid were cultured for 2 days with or without Thiamet G. As shown in Fig 4A, increased protein O-GlcNAcylation induced by Thiamet G (right panel) was associated with a significant increased activity of the U3R-Luc reporter construct compared to untreated cells (left panel), while comparable amount of Tax was produced in each condition (right panel). Importantly, similar results were obtained in HEK-293T cells transfected with the Tax plasmid (Fig 4B).

Hence, enhancing O-GlcNAcylation by pharmacological inhibition of OGA, to mimic the effect of Tax on OGA activity, significantly increases Tax-mediated LTR transactivation.

### Tax increases CREB O-GlcNAcylation

Tax activates the viral LTR via the recruitment of CREB, which has been shown previously to be modified by O-GlcNAcylation [20–22]. This raises the hypothesis that the higher level of LTR transactivation upon OGA inhibition was linked to higher O-GlcNAcylation of CREB. To investigate this point, the impact of Tax on CREB O-GlcNAcylation was studied using capture on wheat germ agarose (WGA), as previously described [30]. HEK-293T cells were transfected or not with the Tax plasmid and were also treated or not with Thiamet G. Two-days after transfection, cells were lysed and same amounts of total proteins were incubated with WGA. O-GlcNAcylated proteins captured on WGA were analyzed by western blot using an anti-O-GlcNAc antibody. As expected, Thiamet G treatment dramatically increased the amount of WGA-bound O-GlcNAcylated proteins (Fig 4C left panel, compare lanes 1 and 3 and quantification on middle panel). Expression of Tax in HEK-293T cells also increased the binding of O-GlcNAcylated protein on WGA (Fig 4C, left panel, compare lanes 1 and 2), albeit at a much lower level than in cells treated with 10 μM Thiamet G. In agreement with this observation, we found that the inhibitory effect of Tax on OGA enzymatic activity in HEK-293T cells corresponds to the inhibitory effect of a much lower concentration of Thiamet G (0.01 μM, S5 Fig). Adding N-acetylglucosamine during incubation of cell lysates with WGA almost completely abolished the anti-O-GlcNAc signal, showing the specificity of the enrichment method (S6 Fig).

Reprobing the membrane with the anti-CREB antibody indicated a massive increase in binding of CREB to WGA upon Thiamet G treatment, confirming CREB as a target of O-GlcNAcylation (Fig 4C, left panel, compare lanes 1 and 3 and quantification on right panel). Expression of Tax also significantly increased CREB retention on WGA, as demonstrated by the higher WGA/lysate ratio for CREB (Fig 4C left panel, compare lanes 1 and 2 and quantification on right panel) suggesting that Tax may induce CREB O-GlcNAcylation. In contrast, Tax was detected in the lysates but not among WGA-bound proteins, neither in absence or presence of Thiamet G. This suggests that Tax does not induce its own O-GlcNAcylation and is unlikely to be an O-GlcNAcylation target, as it is not retained on WGA even in conditions where a major general increase in protein O-GlcNAcylation is induced by pharmacological inhibition of OGA (S7 Fig).

We also evaluated in TL-om1 cells the effect of Tax on CREB retention on WGA (Fig 4D). Same amounts of total proteins were incubated with WGA. Immunodetection were performed on WGA-bound proteins (WGA) or total proteins (Lysates) with either the anti-O-GlcNAc or anti-CREB antibody. First, we observed that the level of total O-GlcNAcylated proteins retained on WGA was higher in lysates from Tax-transfected than in control cells (Fig 4D, left panel and quantification on middle panel). Importantly, the amount of CREB retained on WGA was also higher in Tax-transfected TL-om1 cells than in control cells (Fig 4D, left panel and quantification on right panel).

These data strongly suggest that Tax expression is sufficient to enhance CREB O-GlcNAcylation both in T cells and adherent HEK-293T cells.

### Tax-mediated CREB O-GlcNAcylation on Serine 40 facilitates LTR activation

**Fig 5:**
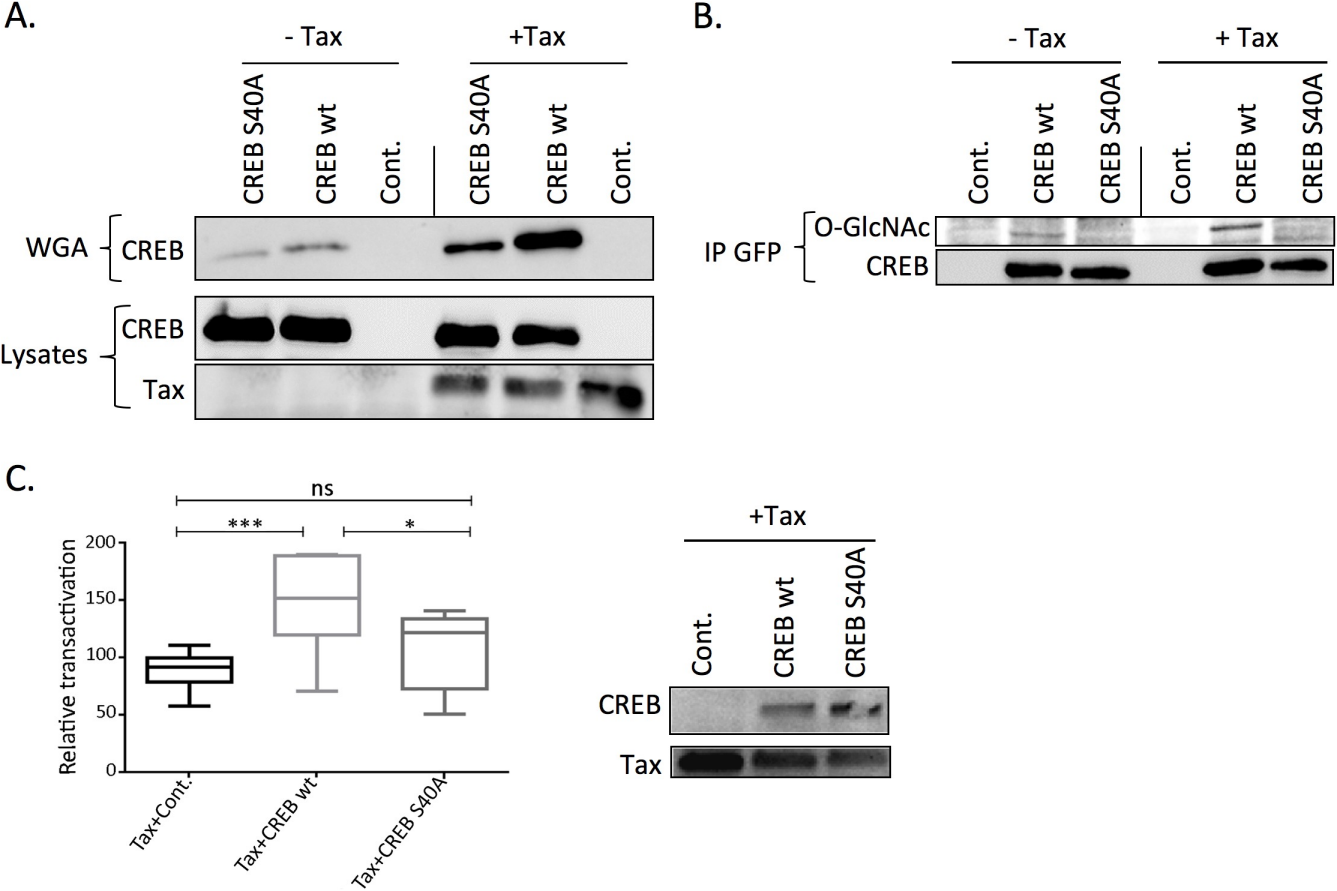
Mutation of CREB Serine 40 inhibits both Tax-mediated increase in CREB O-GlcNAcylation and LTR activation. (**A**) HEK-293T cells were transfected with the control (- Tax) or Tax plasmid (+ Tax) and either the wild-type (wt) or S40A mutant YFP-CREB construct and lysed 48h post-transfection. The amounts of wt and S40A YFP-CREB in the lysate were evaluated by measuring fluorescence emission at 530 nm after excitation at 480 nm. After normalization for fluorescence, O-GlcNAcylated proteins were captured on wheat germ lectin agarose (WGA) beads. Total proteins (Lysates) or WGA-bound proteins (WGA) were separated by SDS-PAGE and blotted with an anti-CREB antibody. Tax expression in cell lysates is also shown. **(B)** HEK-293T cells were transfected and cell extracts were normalized as in (A), but wt and mutant YFP-CREB proteins were immunoprecipitated using an anti-GFP antibody. The level of O-GlcNAcylation of wt and S40A YFP-CREB was evaluated using an anti-O-GlcNAc antibody. The membranes were then striped and reprobed with an anti-CREB antibody. (**C**) Effect of wt or S40A mutant YFP-CREB on Tax-mediated LTR activation. Left panel: HEK-293T cells were transfected with U3R-LTR-luciferase and pRL-TK plasmids along with Tax and either a control plasmid or the plasmid coding for wt or S40A YFP-CREB. Luciferase production was then measured two days post-transfection using the dual luciferase assay. Results are from 4 independent experiments performed in duplicates. Statistical significance was analyzed using the Tukey’s multiple comparison test (*: p<0.05; ***: p<0.001; ns: not significant). Right panel: levels of Tax and CREB expression in the transactivation experiments.

Serine 40 was previously described as a CREB O-GlcNAcylation site [21]. To determine whether Tax induces CREB O-GlcNAcylation on this particular residue, we used YFP-tagged wild-type (wt) and S40A mutant versions of CREB. HEK-293T cells were transfected with Tax and either wt or S40A YFP-tagged CREB and lysed 48 hours post-transfection. After normalization for equivalent amount of YFP-CREB fluorescence, cell lysates were incubated with WGA beads. Western-blotting with the anti-CREB antibody indicated that Tax expression significantly increased the amount of YFP-CREB retained on WGA (Fig 5A). However, residual binding of mutated CREB on WGA suggests that either other O-GlcNAcylation sites still exists on S40A mutant, or that part of this binding occurs through O-GlcNAcylation of some CREB partner.

As a complementary approach, Tax-mediated O-GlcNAcylation of Serine 40 of CREB was analyzed by immunoprecipitation (Fig 5B). Cell lysates from HEK-293T cells transfected or not with Tax and either wt or S40A YFP-tagged CREB were normalized for YFP fluorescence and then immunoprecipitated with an anti-GFP antibody. Western-blotting using the anti-O-GlcNAc antibody revealed that mutation of S40 totally abolished Tax-induced O-GlcNAcylation of CREB, indicating that Serine 40 is indeed the main glycosylation site regulated by Tax. These findings also confirm that WGA binding of CREB mainly depends on O-GlcNAcylation of CREB itself.

We then investigated the effect of expressing either wt or S40A YFP-CREB on Tax-induced LTR transactivation. As expected, transfection of wt YFP-CREB into HEK-293T cells significantly enhanced Tax-mediated transactivation (Fig 5C, left panel). CREB S40A was produced at higher level than wt CREB (Fig 5C, right panel), as previously reported [21]. However, despite this higher expression level, significantly less transactivation was found in cells expressing the S40A mutant than in those producing wt CREB (Fig 5C, left panel).

These findings provide direct evidence that CREB O-GlcNAcylation, especially at Serine 40, is involved in Tax-mediated LTR activation.

### O-GlcNAcylation regulates CREB binding to the LTR

**Fig 6:**
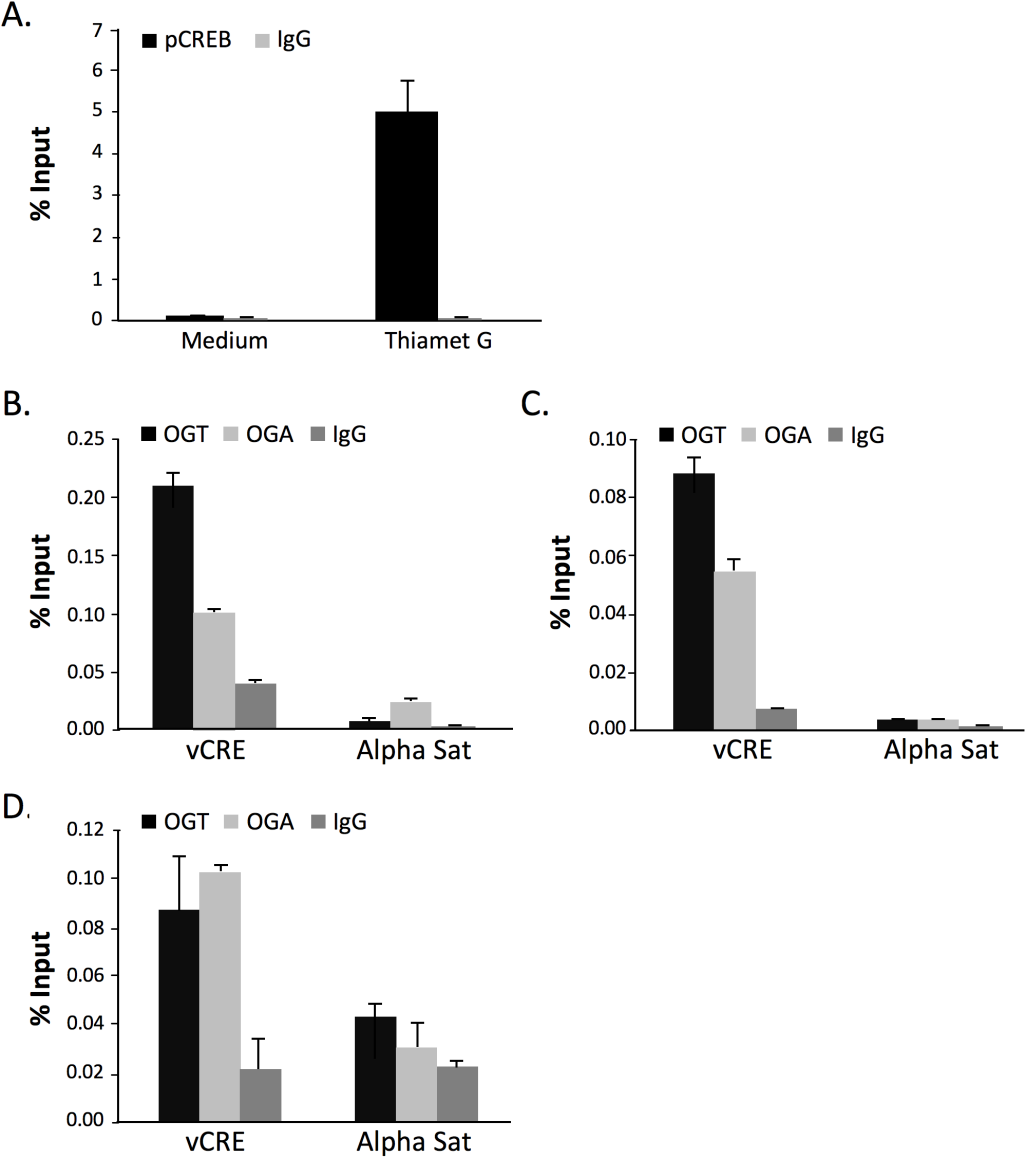
Effect of O-GlcNAcylation on the HTLV-1 LTR. (**A**) Effect of Thiamet G on Ser133 phospho-CREB recruitment to the vCRE LTR region. C8166 T cells were cultured with or without Thiamet G for 48h before chromatin preparation. Chromatin was precipitated with either control IgG or an anti-Ser133 phospho-CREB and recovered DNA was amplified using a pair of primers specific for the vCRE sequence. Results correspond to means ± SEM of triplicate determinations obtained in a representative experiment out of 2. (**B-D**) Detection of OGA or OGT on the vCRE sequence by ChIP in C8166 (**B**) and MT2 (**C**) HTLV-1-transformed T cells or in HTLV-1-immortalized CIB T cells (**D**). Cells were treated as above and chromatin was precipitated using anti-OGT, anti-OGA or control (IgG) antibody. Recovered DNA was amplified using pairs of primers specific for the vCRE sequence or for alpha-satellite sequences as negative control. Results correspond to means ± SEM of triplicate determinations obtained in a representative experiment out of 2.

Since CREB activity on the HTLV-1 LTR is linked to its recruitment to the vCRE regions, we directly analyzed the impact of increasing O-GlcNAcylation on protein recruitment to the vCRE LTR sequences by chromatin immunoprecipitation (ChIP) experiments. As CREB phosphorylated at Serine 133 was shown to be preferentially recruited to the vCRE, ChIP experiments were performed using an anti-phospho CREB (Ser 133) and primers specific for the distal U3 vCRE sequence. Thiamet G treatment of C8166 T cells dramatically increased the amount of amplified vCRE products as compared to untreated cells (Fig 6A).

Whether the O-GlcNAczyme complex was also recruited to the vCRE region was finally investigated by ChIP in C8166 T cells. Both anti-OGT and anti-OGA ChIP allowed the amplification of vCRE-specific products to levels significantly higher than the control IgG (Fig 6B). Moreover, very low amplification signals were detected with primers targeting alpha-satellite (alpha-sat) regions, showing the specificity of the anti-OGT and anti-OGA ChIPs. Importantly, similar results were obtained with another HTLV-1-transformed T cell line (MT2, Fig 6C) as well as with HTLV-1-immortalized T cells (CIB, Fig 6D).

Hence, CREB recruitment to the LTR is facilitated by O-GlcNAcylation and the OGT/OGA O-GlcNAczyme complex is recruited to the vCRE sequences of the HTLV-1 LTR.

## Discussion

The HTLV-1 Tax oncoprotein is critical for both HTLV-1 expression and HTLV-1-mediated T-cell immortalization. Therefore, the characterization of activators or co-factors responsible for the transactivation of the HTLV-1 5’ LTR is an important issue. In this study, we provide the first demonstration that a novel molecular actor, the O-GlcNAczyme complex, interacts with Tax and is recruited to the LTR as a positive co-factor in both HTLV-1 immortalized and transformed T cells.

We first documented that HTLV-1-transformed T cells express higher level of OGA than control transformed T cells but that this coincides with a dramatic reduction in the specific activity of OGA. Furthermore, expressing only Tax was sufficient to inhibit OGA activity (Fig 2A and 2D) and to increase O-GlcNAcylation of a BRET-based biosensor (Fig 2C and 2E) in both Tax-negative HTLV-1-transformed TL-om1 T cells and HTLV-1-negative HEK-293T cells. This suggests that the ability of Tax to inhibit OGA and increase O-GlcNAcylation is independent of HBZ, as this inhibition is observed in HEK-293T cells which do not express any HTLV-1 protein. However, a potential blocking effect of HBZ on Tax-induced inhibition of OGA activity cannot be ruled-out and should be investigated in future studies. The inhibitory effect of Tax appeared to be specific for OGA activity, as OGT enzymatic activity was not affected by Tax transfection (S3 Fig).

Increased OGA expression associated with impaired OGA activity is not unprecedented. Indeed, previous studies reported that pharmacological inhibition of OGA also leads to OGA accumulation, presumably as the result of a regulatory feedback mechanism compensating for loss of enzymatic activity [31, 32]. We propose therefore that the increased OGA expression found in HTLV-1 transformed T cells is an adaptive response, operating through a yet unknown mechanism, to counteract the inhibition of OGA activity by Tax.

Previous studies have indicated that OGA and OGT associate into a molecular assembly denominated O-GlcNAczyme [23]. Using BRET experiments, we found on the one hand that Tax interacts with both OGT and OGA (Fig 3A), and on the other hand, that Tax expression significantly increases the affinity between OGT and OGA (Fig 3C). This was associated with a significant reduction of OGA enzymatic activity in the complex (Fig 3D). Further experiments are needed to unravel the mechanism by which Tax regulates OGA activity within the O-GlcNAczyme complex, resulting in increased protein O-GlcNAcylation.

Our data also provided evidence that an important consequence of Tax-induced OGA inhibition is the higher O-GlcNAcylation of CREB. Indeed, we showed that expressing Tax in either HEK-293T cells or TL-om1 T cells significantly increased the amount of WGA-bound CREB (Fig 4C and 4D). Strikingly, higher WGA-bound CREB was also found upon pharmacological inhibition of OGA by Thiamet G, and Tax expression did not further increase CREB binding to WGA in Thiamet G-treated cells (Fig 4C). This confirms that Tax effect on O-GlcNAcylation is mediated by inhibition of OGA, as it is not anymore detectable in cells in which OGA activity is maximally inhibited by the drug. O-GlcNAcylation of CREB was confirmed in experiments showing that CREB binding to WGA was markedly reduced by mutation of Serine 40, a previously identified O-GlcNAcylation site on CREB (Fig 5A). Importantly, the effect of Tax on O-GlcNAcylation of CREB was directly shown by immunoprecipitation of CREB followed by western-blotting using anti-O-GlcNAc antibody (Fig 5B). Moreover, these experiments demonstrated that Tax induced O-GlcNAcylation of wt but not of S40A CREB (Fig 5B).

Regarding the functional impact of CREB O-GlcNAcylation, we found that treating cells with the selective OGA inhibitor Thiamet G increased Tax-mediated LTR transactivation both in C8166 T cells and adherent HEK-293T cells (Fig 4A and 4B). Moreover, Thiamet G treatment strongly enhanced the recruitment of Ser133-phosphorylated CREB to the vCRE region of the LTR (Fig 6A). This finding suggests that CREB O-GlcNAcylation and phosphorylation are not mutually exclusive, in agreement with a previous report [21]. Hence, our results indicate that OGA inhibition upon Tax expression or Thiamet G treatment increases CREB O-GlcNAcylation and thereby its activity on the LTR. In agreement with this hypothesis, we showed that Tax-mediated transactivation was enhanced upon expression of wt CREB but not of O-GlcNAcylation-defective S40A CREB mutant (Fig 5C), directly linking CREB O-GlcNAcylation, notably at Serine 40, and Tax-induced LTR activation. Interestingly, O-GlcNAcylation of CREB at Serine 40 was previously shown to block CREB transcriptional activity in neuronal cells by preventing CREB association with CRTC [21]. In contrast, we report here that CREB S40A is impaired for Tax-mediated transactivation, indicative of an activating role of this O-GlcNAcylation site in our model. This suggests that interaction with CRTC required for CREB function in neuronal cells is not a key determinant in the case of Tax-mediated LTR activation. CRTC/TORC was described as a coactivator of Tax-mediated LTR transactivation [14, 33]. However, Siu and collaborators showed that silencing all three CRTC/TORC family members only partially reduced Tax-mediated LTR activation [14], indicating that Tax can still activate the LTR without CRTC/TORC. Moreover, ATF4/CREB2, which does not need CRTC as coactivator [14], is able to activate the LTR in presence of Tax [34, 35]. Hence, depending of the promoter context, CREB O-GlcNAcylation at Serine 40 may mediate either activating or repressive functions. Interestingly, such opposite effect of O-GlcNAcylation has previously been reported for other transcription factors, notably RelA and Sp1 [19, 36–40].

OGT is now considered as an epigenetic regulator by virtue of its capacity to add O-GlcNAcylation on epifactors and histones (reviewed in [26, 41]). Consequently, OGT binding to promoters has been described [42]. We show here that not only OGT but also OGA are recruited to the vCRE region of the HTLV-1 LTR, suggesting the presence of the O-GlcNAczyme complex at the promoter and its involvement in HTLV-1 gene regulation. Our data therefore support a model in which, *via* the deposition of the O-GlcNAczyme complex onto the vCRE region, Tax facilitates the O-GlcNAcylation of CREB and possibly other transcription factors and co-factors while concomitantly modulating local chromatin architecture. This ultimately increases promoter activation, as documented here by the positive effect of OGA inhibition on the transactivation by Tax of the HTLV-1-U3R-Luc reporter construct. Importantly, recruitment of OGT and OGA to the 5’LTR was found in both HTLV-1-immortalized primary T cell and HTLV-1-transformed T cell lines. This provides the notion that the O-GlcNAczyme complex may play a key role in both HTLV-1-replication *in vivo* and HTLV-1-induced pathologies.

Only few studies have investigated the impact of O-GlcNAcylation on virus transcription. It has been shown that enhancing O-GlcNAcylation inhibits the expression of human immunodeficiency virus type 1 or herpes virus simplex [43, 44]. In these cases, the effect was linked to the modification of transcriptional regulators, Sp1 and HCF-1 respectively, involved in virus expression. Our data showing that O-GlcNAcylation increases the transactivation of the HTLV-1 LTR provide therefore the first example of a positive impact of the O-GlcNAcylation machinery on viral transcription via the recruitment of the O-GlcNAczyme complex to the viral promoter.

## Materials and methods

### Cells and transfections

HEK-293T cells (American Type Culture Collection CRL-3216) were grown in Dulbecco’s modified Eagle’s medium supplemented with 10% fetal calf serum (Dutcher, S1402851810) and with 2 mM glutamine, 1 mM pyruvate and antibiotics (Invitrogen) and were transfected using the Fugene 6 reagent (Invitrogen). The non-infected CD4+ T-cell lines Jurkat (kindly provided by Dr. Schwartz, Institut Pasteur, Paris, France), Molt4 (American Type Culture Collection CRL-1582), CEM (American Type Culture Collection CRL-1992) and HUT-78 (American Type Culture Collection TIB-161) and the HTLV-1-transformed CD4+ T-cell lines C8166 and MT2 (NIH AIDS Research and Reference Reagent Program, USA) and TL-om1 (kindly provided by Dr. Harhaj, Johns Hopkins School of Medicine, Baltimore, USA) were grown in RPMI 1640 medium containing 25mM glucose and supplemented as above but with the addition of 20 mM HEPES and 5mL of 100X non-essential aminoacid solution (Invitrogen). TL-om1 T cells were transfected by nucleofection using the cell line nucleofector kit V (Lonza, France) and the program O-017. The HTLV-1-immortalized CIB T cells described in [45] were generated from peripheral blood mononuclear cells of a TSP/HAM patient. These cells were grown in supplemented RPMI medium in the presence of 50U/ml of IL-2 (Roche, France).

### Plasmids

The pSG5M empty vector, pSG5M-Tax and pRL-TK plasmids have been described elsewhere [46]. The U3R-Luc construct described in [47] was kindly provided by Dr. A. Kress (Erlangen, Germany). The YFP-CREB wild-type plasmid was kindly provided by Prof. Montminy (La Jolla, USA). YFP-CREB S40A was generated by PCR-mediated mutagenesis using the following primers: Fw: TGCCACATTAGCCCAGGTAgCCATGCCAGCAGCTCATG and Rev: CATGAGCTGCTGGCATGGcTACCTGGGCTAATGTGGCA and the presence of the mutation was verified by sequencing. The pcDNA3Rluc8 plasmid was a kind gift of Prof. Gambhir [48]. The pcDNA3.1 Rluc8-Tax plasmid was generated following PCR amplification of the Tax sequence from the pSG5M-Tax vector using primers creating NheI restriction sites at both extremities of Tax cDNA (forward: GGCGCTAGCCACCATGGCCCACTTCCCAGGG; reverse: GCCGCTAGCTCCGA-CTTCTGTTTCTCGGAAATG). The PCR product was then inserted into the pcDNA3.1 RLuc8 after NheI digestion. YFP-OGA has been described previously [30]. Rluc8-OGT was generated by inserting OGT coding sequence [49] into the pcDNA3.1 Rluc8 vector after HinDIII/ BamHI digestion. YPet-OGT was obtained by insertion of cDNA OGT sequence into YPet-pcDNA3 vector after digestion with EcoRV-Apa1.

To monitor O-GlcNAcylation in living cells, we developed a BRET-biosensor based on the previously described FRET OS2-O-GlcNAc biosensor [27] by replacing the CFP by an Rluc8 sequence. The BRET biosensor is composed of Rluc8 fused to the fimbrial adhesin lectin domain GafD, a known OGT substrate peptide derived from casein kinase II placed between two flexible linkers (GGSGG) followed by a variant of the yellow fluorescent protein Venus (Fig 2B).

### Antibodies and reagents

Tax was detected using sera from HTLV-1 infected individuals (kindly provided by Dr Gessain, Institut Pasteur, Paris, France) or the anti-Tax monoclonal antibody (mab) 168-A51 (NIH AIDS Research and Reference Reagent Program, USA). The following primary antibodies were used: anti-GFP recognizing GFP as well as the YFP and YPET variants (Roche Applied Science), anti-OGT (Sigma, DM-17 06264), anti-OGA (Santa Cruz, sc135093 or Sigma, SAB4200311), anti-O-GlcNAc (Abcam, RL2), anti-CREB (Millipore, CS 203204), anti-phospho CREB ser133 (Millipore, CS 204400), anti-actin (Santa Cruz, sc1616), anti-tubulin (GeneTex, GT114) and GAPDH (Santa Cruz, sc32233). HRP-conjugated anti-human, anti-mouse and anti-rabbit IgG (Promega) were used as secondary antibodies. Thiamet G (Sigma, SML 0244) was used at 10μM concentration.

### Luciferase assays

C8166 T cells (2x10^6^/12 well in duplicates) were cotransfected by nucleofection with 700 ng of the U3R-Luc reporter plasmid and 200 ng of the Renilla reporter plasmid pRL-TK. 293T cells seeded in duplicates in 24-well (3x10^4^/well) were co-transfected with 500 ng of the U3R-Luc plasmid and 50 ng of pRL-TK, and with 500 ng of the control or the Tax plasmids with or without 200 ng of the YFP-CREB constructs. Luciferase activity was determined using the Dual Luciferase Assay System (Promega) and values were normalized with Renilla activity.

### Cell lysis, immunoprecipitation, wheat germ lectin precipitation and immunoblot

Cells were lysed in lysis buffer (50 mM Tris-HCl pH8, 1% NP40, 0.5% deoxycholate, 0.1% SDS and 150 mM NaCl) supplemented with protease and phosphatase inhibitors (Roche). Immunoprecipitations were carried out as follow: cell lysates were incubated overnight with primary antibodies at 4°C, and antibody complexes were captured on protein G-sepharose beads (GE Healthcare) 1h at 4°C. Sepharose beads were then washed 5 times in washing buffer (120 mM NaCl, 20mM Tris-HCl pH8, 0.2 mM NaF, 0.2 mM EGTA, 0.2% deoxycholate, 0.5% NP40) before elution in Laemmli buffer. O-GlcNAcylated proteins were precipitated on 40 μL of WGL-agarose (WGA) beads (Vector Laboratories, Paris, France) for 2h at 4°C. WGA beads were then washed 5 times in washing buffer and captured proteins then eluted in Laemmli buffer as described in [50]. In some experiments, N-acetylglucosamine (500 mM) was added during incubation with WGA beads as a control for non-specific binding of protein to WGA. Immunoprecipitated, WGA-precipitated proteins, and total cell lysates were separated by SDS-PAGE, transferred to membranes and blotted with specific antibodies.

### RNA extraction and qRT-PCR

Total RNAs were prepared with the Nucleospin RNAII kit (Macherey Nagel, France) and 1μg of RNA was reverse transcribed using the Maxima first strand cDNA synthesis kit (Thermo Scientific, France), according to the manufacturer’s procedure. Real-time-PCR was performed in the Lightcycler 2.0 (Roche, France) on 10 ng of reverse transcribed RNA using the following primers: OGT (forward: GCCCTGGGTCGCTTGGAAGA, reverse: TGC CAC AGC TCT GTCAAAAA), OGA (forward: TCTGCGGTGTGGTGGAAGGA, reverse: TGGGGTTAGAAAAAGTGATA) and the housekeeping gene HPRT (forward: 5’TGACACTGGCAAAACAATGCA3’, reverse: 5’GGTCCTTTTCACCAGCAAGCT3’) for normalization. PCR was conducted using the Sybr Green method with the following conditions: a first step of denaturation at 95°C for 8 min, followed by 40 cycles of denaturation (95°C for 10 sec), annealing (60°C for 10 sec), and extension (72°C for 8 sec) and a final step of melting curve (95°C for 5 sec, then 65°C for 15 sec. and finally 95°C for 10 sec).

### CHIP experiments

Before the experiment, 10^7^ C8166, MT2 or CIB cells were crosslinked using first 0,08% Disuccinimidylglutarate (SantaCruz Biotechnologies) during 30 min at room temperature and 1% Formaldehyde (Electron Microscopy Sciences) for 10 minutes at room temperature. Chromatin was then sheared using a Bioruptor Pico sonicator to obtain fragments of around 300 bp. Ten μg of chromatin were used for each condition. ChIP experiments were performed using the ChIP-IT high sensitivity kit from active motif. Primer pairs that specifically amplify the distal vCRE (position 201–275: Forward 5’ATCATAAGCTCAGACCTCCGGGAA3’, reverse 5’CCTGAGGACGGCTTGACAAACAT3’) were used for PCR.

### BRET measurements

HEK-293T cells were transfected in 12 well plates as described previously [51] using 300 ng of each cDNA construct, unless otherwise stated in the figure legend. One day after transfection, cells were transferred into 96-well microplate, and BRET measurements were carried out on the following day. TL-om1 T cells were transfected by nucleofection in 12 well plates. On the following day, cells were distributed into 96 well microplate and BRET measurements were performed.

For BRET measurements, cells were pre-incubated for 5 min in PBS in the presence of 5 μM coelenterazine. Light-emission acquisition at 485 nm and 530 nm was then started, and signal acquisition was performed every minute during 20–30 min using TECAN Infinite F200 Pro apparatus. BRET signal was expressed in milliBRET units (mBU). The BRET unit has been defined previously as the ratio 530 nm/485 nm obtained when the two partners are present, corrected by the ratio 530 nm/485 nm obtained under the same experimental conditions, when only the partner fused to Renilla luciferase is present in the assay [52]. Each measurement corresponded to the signal emitted by the whole population of cells present in a well (i.e., approximatively 4x10^4^ HEK 293 cells or 10^6^ TL-om1 T cells).

### OGA and OGT activities

OGA activity was measured using 4-methylumbellifery-N-acetylβ-D-glucosamine (MU-GlcNAc, Sigma), which is converted into fluorescent 4-methylumbelliferon upon hydrolysis by OGA and other hexosaminidase [53]. 4-methylumbelliferon fluorescence was measured at 448 nm after excitation at 362 nm after 30 min and 60 min incubation at 37°C, to ensure that the determination was performed during the linear phase of the reaction. To determine the concentration of 4-methylumbelliferon, a standard curve was performed in each experiment using commercial 4-methylumbelliferon (Sigma). To specifically determine OGA activity versus other glycosydases, all reactions were performed in absence or presence of the highly specific OGA inhibitor Thiamet G. The difference of the fluorescent signal obtained in absence and presence of Thiamet G reflected the amount of 4-methylumbelliferon produced by OGA.

To measure OGT activity, OGT was immunoprecipitated using an anti-OGT antibody (Sigma-Aldrich) for 2h at 4°C. Precipitation was performed by incubating 50μL equilibrated protein G-sepharose beads (GE Healthcare) for 30 min at 4°C. After 3 washes, the precipitated proteins were submitted to an additional wash in OGT assay buffer containing 50 mM Tris-HCl and 12.5 mM MgCl2, pH7.5 and 1μM Thiamet G. OGT assay was then performed on protein-G sepharose bound OGT using the bioluminescent UDP-GloTM glycosyltransferase assay (Promega) exactly as described in the manufacturer instructions [54].

### Ethics statement

The use of peripheral blood mononuclear cells from patient CIB was approved by the French Comité Consultatif de Protection des Personnes dans la Recherche Biomédicale (CCPPRB) and the patient provided a written informed consent.

## Supporting information

S1 Fig Effect of T-cell activation on the expression of OGT and OGA transcripts. Peripheral blood mononuclear cells (PBMC) from 2 different donors were stimulated with phytohemagglutinin (1 μg/mL) in the presence of interleukin 2 (50 U/mL) and cultured for 3 days. The levels of OGT and OGA transcripts were then quantified by RT-qPCR and normalized to the level of the housekeeping gene HPRT. Results are the means of triplicates determination for each donor.
(PDF)

S2 Fig Linear relationship between the amount of OGA quantified by western-blot and the level of OGA activity measured by the fluorescent assay. HEK-293T cells were lysed and protein concentration in the lysate was determined. Increasing amounts of total proteins were loaded on a SDS-PAGE for evaluation of OGA expression level by western-blotting using an anti-OGA antibody. In parallel, OGA activity was measured using the 4-methylumbellifery-N-acetylβ-D-glucosamine fluorescent assay. The signal obtained by densitometric analysis of the 130 kDa band was plotted against OGA activity and analysed using Pearson correlation analysis.
(PDF)

S3 Fig Tax expression does not affect OGT activity in HEK-293T cells. HEK-293T cells were transfected with either the Tax or control plasmid. 48h after transfection, cells were lysed and OGT was immunoprecipitated using an anti-OGT antibody. The enzymatic activity was measured on OGT bound to protein-G sepharose using the bioluminescent UDP-GloTM glycosyltransferase assay (Promega). Results are the mean ± SEM of 3 independent experiments and are expressed as fold effect of the control condition (pSG5M transfected cells). Statistical analysis was performed using a t test for unpaired values (ns: not significant).
(PDF)

S4 Fig Expression of the proteins used in the BRET assay. HEK-293T cells plated in 12-well plates were co-transfected with Rluc8-Tax and either YFP-OGA or YPET-OGT. Protein expression was analyzed by western blot 48h after transfection. Proteins were detected using an anti-Tax or anti-GFP (which also recognizes the YFP or YPET variants) antibody. Given the molecular weight of Tax (40 kDa), Rluc8 (37 kDa), YFP/YPET (27 kDa), OGA (130 kDa) and OGT (110 kDa) the expected molecular weight of Rluc8-Tax, YFP-OGA or YPET-OGT are 77 kDa, 157 kDa and 137 kDa, respectively.
(PDF)

S5 Fig Comparison of OGA inhibition by Thiamet G and by Tax expression in HEK 293 T cells. To compare the potency of Tax inhibition to that of Thiamet G, a dose-response of Thiamet G effect on OGA activity was performed. OGA assay was performed as described in the method section using HEK 293-T cell lysates (30 μg of proteins), in absence or presence of increasing concentrations of Thiamet G. For comparison of these data with the effect of Tax on OGA activity in HEK-293T (shown in Fig 2D), basal OGA activities in the two experiments were set at 100%. The inhibitory effect obtained with Tax transfection on OGA activity measured on the same amount of protein lysate was similar to the inhibitory effect obtained with 0.01 μM Thiamet G (about 60% of residual activity).
(PDF)

S6 Fig N-acetylglucosamine blocks binding of O-GlcNAcylated proteins to WGA. HEK-293T cells were transfected with either the control or Tax plasmid and treated or not with Thiamet G and cell extracts were prepared two days post-transfection. Cell lysates were incubated with WGA beads in presence or absence of 500 mM of N-acetylglucosamine (which competes with O-GlcNAcylated proteins for WGA binding). Proteins were then separated by SDS-PAGE and blotted with an anti-O-GlcNAc specific antibody (RL2).
(PDF)

S7 Fig Tax is not detected among WGA-bound proteins in transfected HEK-293T cells. HEK-293T cells were transfected with either the control or Tax plasmid and treated or not with Thiamet G, and cell extracts were prepared two days post-transfection. O-GlcNAcylated proteins were purified via binding to wheat germ lectin agarose beads (WGA), separated by SDS-PAGE and blotted with either an anti-O-GlcNAc or anti-Tax antibody. Tax could be readily detected in lysates from Tax transfected cells while it is not detectable in WGA eluates.
(PDF)
