# Supplementary figures and images for: Hijacking of the O-GlcNAcZYME complex by the HTLV-1 Tax oncoprotein facilitates viral transcription
Source: PLoS Pathog. 2017 Jul 24;13(7):e1006518. doi: 10.1371/journal.ppat.1006518 (PMC5542696; doi:10.1371/journal.ppat.1006518)

S1 Fig

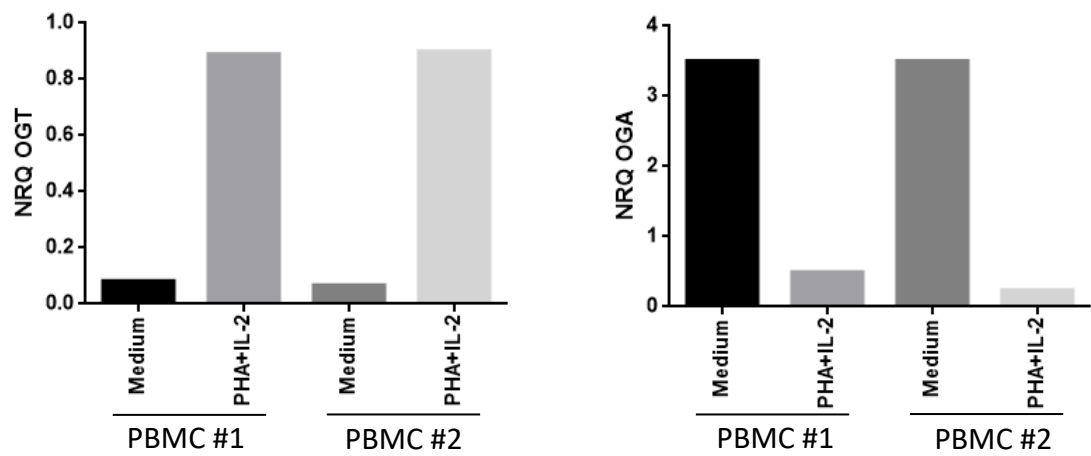

Supplement: S1 Fig — Peripheral blood mononuclear cells (PBMC) from 2 different donors were stimulated with phytohemagglutinin (1 μg/mL) in the presence of interleukin 2 (50 U/mL) and cultured for 3 days. The levels of OGT and OGA transcripts were then quantified by RT-qPCR and normalized to the level of the housekeeping gene HPRT. Results are the means of triplicates determination for each donor. (PDF) [file ppat.1006518.s001.pdf]

S2 Fig

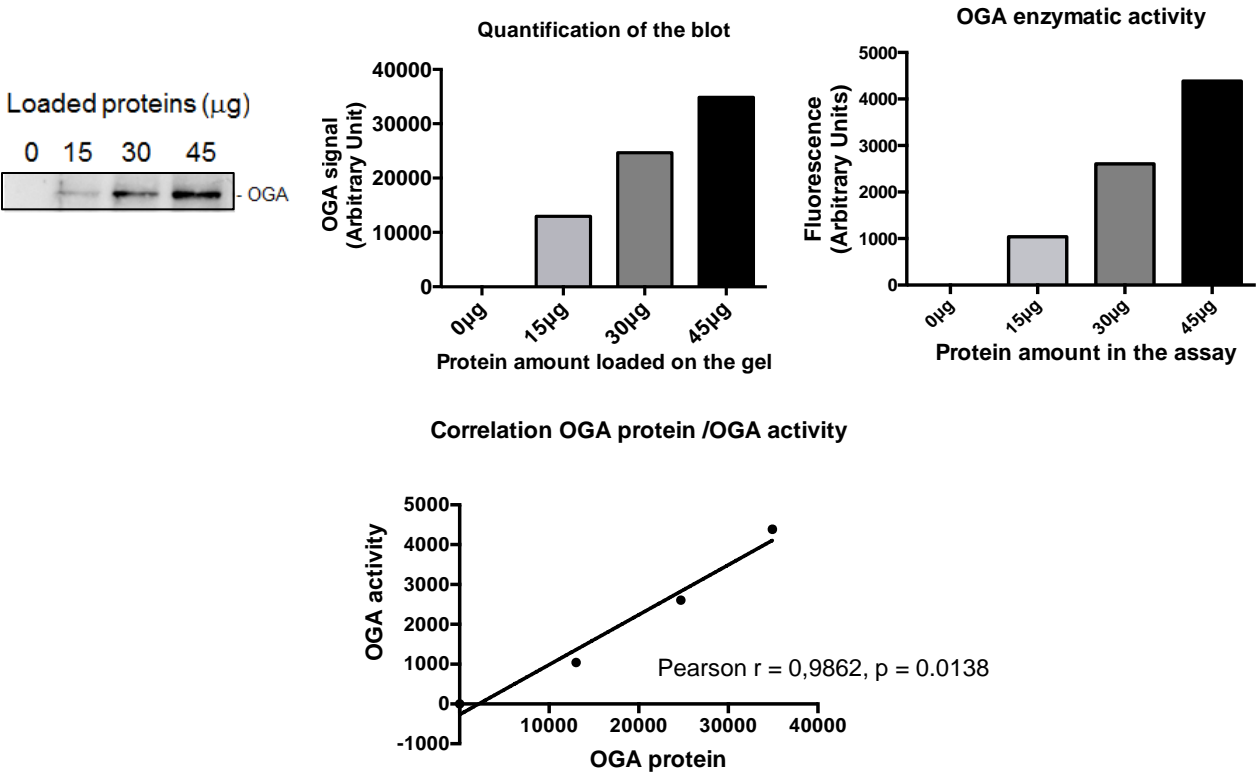

Supplement: S2 Fig — HEK-293T cells were lysed and protein concentration in the lysate was determined. Increasing amounts of total proteins were loaded on a SDS-PAGE for evaluation of OGA expression level by western-blotting using an anti-OGA antibody. In parallel, OGA activity was measured using the 4-methylumbellifery-N-acetylβ-D-glucosamine fluorescent assay. The signal obtained by densitometric analysis of the 130 kDa band was plotted against OGA activity and analysed using Pearson correlation analysis. (PDF) [file ppat.1006518.s002.pdf]

**S3 Fig**

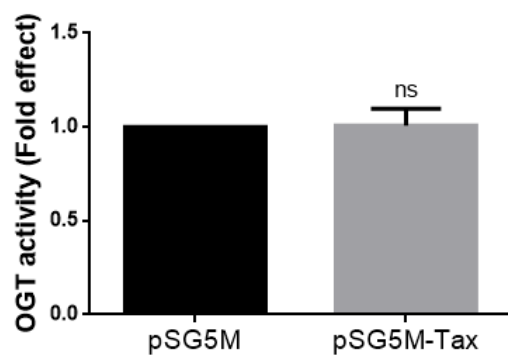

Supplement: S3 Fig — HEK-293T cells were transfected with either the Tax or control plasmid. 48h after transfection, cells were lysed and OGT was immunoprecipitated using an anti-OGT antibody. The enzymatic activity was measured on OGT bound to protein-G sepharose using the bioluminescent UDP-GloTM glycosyltransferase assay (Promega). Results are the mean ± SEM of 3 independent experiments and are expressed as fold effect of the control condition (pSG5M transfected cells). Statistical analysis was performed using a t test for unpaired values (ns: not significant). (PDF) [file ppat.1006518.s003.pdf]

**S4 Fig**

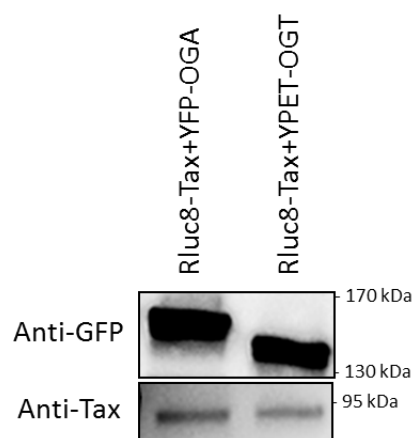

Supplement: S4 Fig — HEK-293T cells plated in 12-well plates were co-transfected with Rluc8-Tax and either YFP-OGA or YPET-OGT. Protein expression was analyzed by western blot 48h after transfection. Proteins were detected using an anti-Tax or anti-GFP (which also recognizes the YFP or YPET variants) antibody. Given the molecular weight of Tax (40 kDa), Rluc8 (37 kDa), YFP/YPET (27 kDa), OGA (130 kDa) and OGT (110 kDa) the expected molecular weight of Rluc8-Tax, YFP-OGA or YPET-OGT are 77 kDa, 157 kDa and 137 kDa, respectively. (PDF) [file ppat.1006518.s004.pdf]

S5 Fig

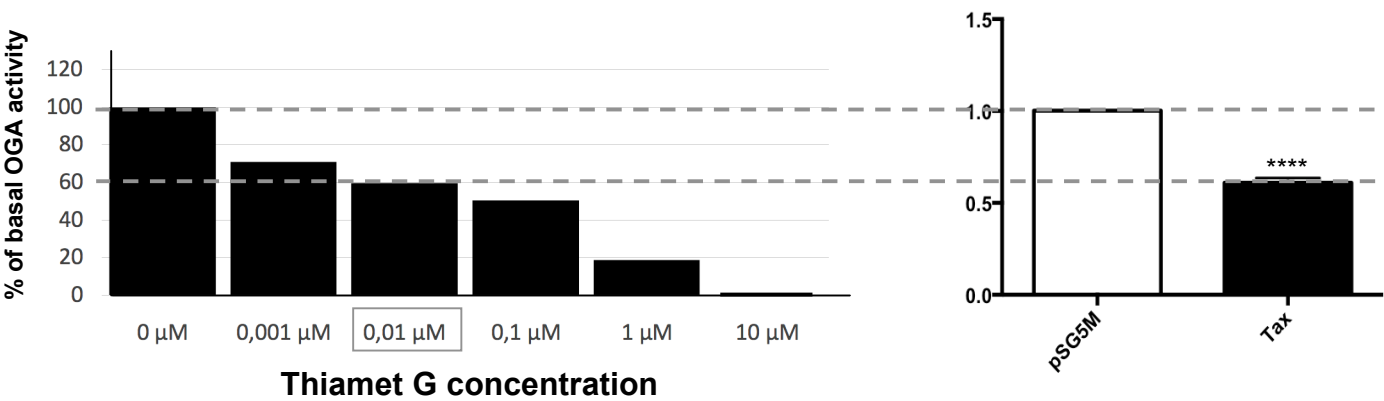

Supplement: S5 Fig — To compare the potency of Tax inhibition to that of Thiamet G, a dose-response of Thiamet G effect on OGA activity was performed. OGA assay was performed as described in the method section using HEK 293-T cell lysates (30 μg of proteins), in absence or presence of increasing concentrations of Thiamet G. For comparison of these data with the effect of Tax on OGA activity in HEK-293T (shown in Fig 2D), basal OGA activities in the two experiments were set at 100%. The inhibitory effect obtained with Tax transfection on OGA activity measured on the same amount of protein lysate was similar to the inhibitory effect obtained with 0.01 μM Thiamet G (about 60% of residual activity). (PDF) [file ppat.1006518.s005.pdf]

S6 Fig

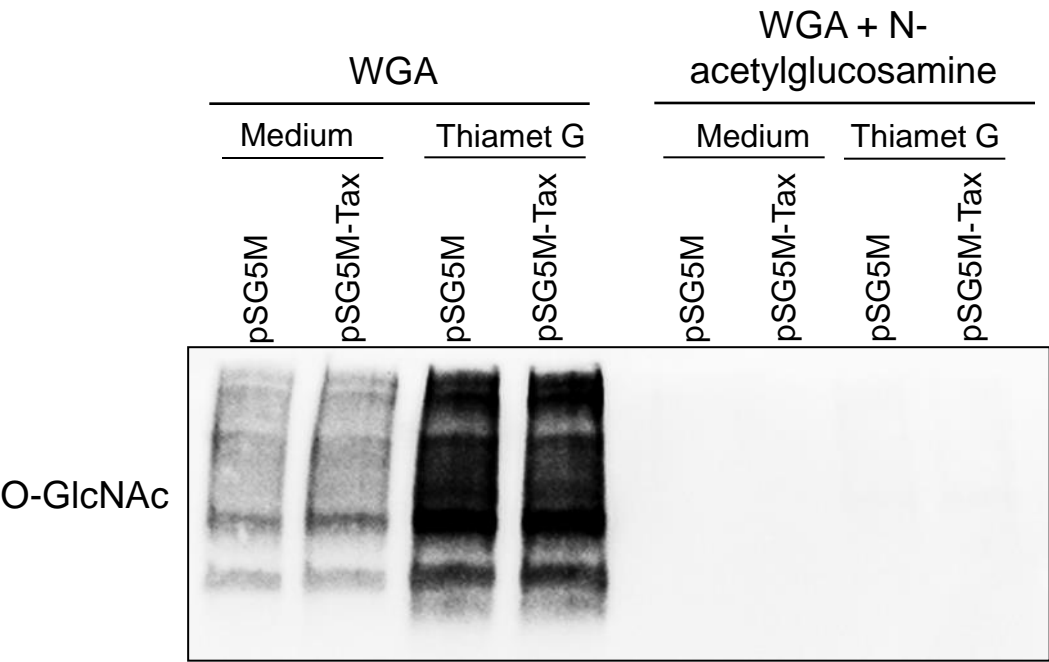

Supplement: S6 Fig — HEK-293T cells were transfected with either the control or Tax plasmid and treated or not with Thiamet G and cell extracts were prepared two days post-transfection. Cell lysates were incubated with WGA beads in presence or absence of 500 mM of N-acetylglucosamine (which competes with O-GlcNAcylated proteins for WGA binding). Proteins were then separated by SDS-PAGE and blotted with an anti-O-GlcNAc specific antibody (RL2). (PDF) [file ppat.1006518.s006.pdf]

S7 Fig

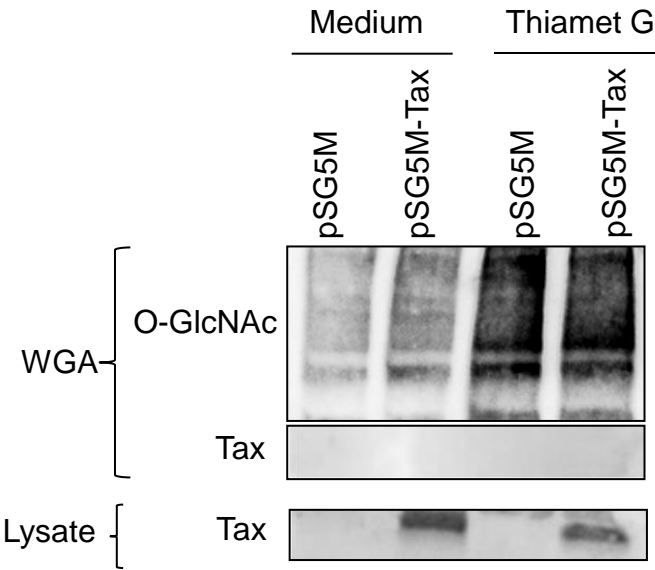

Supplement: S7 Fig — HEK-293T cells were transfected with either the control or Tax plasmid and treated or not with Thiamet G, and cell extracts were prepared two days post-transfection. O-GlcNAcylated proteins were purified via binding to wheat germ lectin agarose beads (WGA), separated by SDS-PAGE and blotted with either an anti-O-GlcNAc or anti-Tax antibody. Tax could be readily detected in lysates from Tax transfected cells while it is not detectable in WGA eluates. (PDF) [file ppat.1006518.s007.pdf]
